# Methylenetetrahydrofolate Reductase (MTHFR) Polymorphisms and Susceptibility for Cervical Lesions: A Meta-Analysis

**DOI:** 10.1371/journal.pone.0052381

**Published:** 2012-12-21

**Authors:** Shuyu Long, Xingliang Yang, Xiaojiao Liu, Pei Yang

**Affiliations:** 1 Department of Gynecology and Obstetrics, West China Second Hospital, Sichuan University, Chengdu, Sichuan, China; 2 Department of Urology, Second Affiliated Hospital, Third Military Medical University, Chongqing, China; University of Nebraska Medical Center, United States of America

## Abstract

**Background:**

The association between the methylenetetrahydrofolate reductase (MTHFR) C677T/A1298C polymorphisms and the susceptibility to cervical lesions was unclear. This study was designed to investigate their precise association using a large-scale meta-analysis.

**Methods:**

The previous 16 studies were identified by searching PubMed, Embase and CBM databases. The crude odds ratios and their corresponding 95% confidence intervals (CIs) were used to estimate the association between the MTHFR C677T/A1298C polymorphisms and the susceptibility to the cervical lesions. The subgroup analyses were made on the following: pathological history, geographic region, ethnicity, source of controls and source of DNA for genotyping.

**Results:**

Neither of the polymorphisms had a significant association with the susceptibility to the cervical lesions in all genetic models. Similar results were found in the subgroup analyses. No association was found between the MTHFR C677T polymorphism and the cervical lesions in the Asia or the America populations though a significant inverse association was found in the Europe population (additive model: *P* = 0.006, OR = 0.83, 95% CI = 0.72–0.95; CT *vs*. CC: *P* = 0.05, OR = 0.83, 95% CI = 0.69–1.00; TT *vs*. CC: *P* = 0.05, OR = 0.73, 95% CI = 0.53–1.00). Interestingly, women with the MTHFR A1298C polymorphisms had a marginally increased susceptibility to invasive cancer (ICC) when compared with no carriers but no statistically significant difference in the dominant model (*P* = 0.06, OR = 1.21, 95% CI = 0.99–1.49) and AC *vs*. AA (*P* = 0.09, OR = 1.21, 95% CI = 0.97–1.51).

**Conclusions:**

The MTHFR C677T and A1298C polymorphisms may not increase the susceptibility to cervical lesions. However, the meta-analysis reveals a negative association between the MTHFR C677T polymorphisms and the cervical lesions, especially in the European populations. The marginal association between the MTHFR A1298C polymorphisms and the susceptibility to cervical cancer requires a further study.

## Introduction

Cervical cancer is the third most frequently encountered cancer and the fourth leading cause of the women’s cancer death in the world, accounting for 9% (529,800) of the total newly-diagnosed cancer cases and 8% (275,100) of the total cancer deaths among females in 2008 [1]. However, cervical cancer is considered a preventable disease because of its relatively long period of precancerous lesions, including cervical intraepithelial neoplasia (CIN). The virological, molecular, clinical and epidemiological studies have provided evidence that cervical cancer is in fact a sequel to a long-term unresolved infection of certain genotypes of the Human Papilloma Virus (HPV) [2], [3]. High-risk HPVs are known to infect cervical epithelium, with a subset of these being associated with preneoblastic lesions that can progress to cervical cancer. Nevertheless, despite the extremely high rate of infection by these viruses, the rate of cervical cancer, even in the prescreening area, has been less than one tenth that of exposure [4], [5]. Thus, other factors are important for cervical lesion development and progression such as a long-term use of hormonal contraceptives, multiparty, smoking, and some nutritional factors [6]–[8].

Association between micronutrient depletion, particularly folate deficiency, and cervical lesions has been studied for a long time. Folate deficiency, as a potential risk for cervical cancer, was first reported by some cytopathologists in the 1960s, who had found that the cervical epithelial cells from folate-deficient women had some similarity to the dysplastic cervical cells in cytology [9]. Later on, Whitehead et al. demonstrated that macrocytic changes in the cervical cells of the oral contraceptive users could be reversed with folic acid supplementation [10]. However, conflicting results still existed in the conclusion of the association between the folate deficiency and the cervical dysplasia [11]–[13]. Furthermore, various clinical epidemiological studies have shown that low-level folate was not directly increase risk of cervical dysplasia but enhance HPV infection instead [14]–[16]. Therefore, despite the lack of a statistically significant association between folate status and cervical dysplasia, these trials indicated that folate may involve along with HPV to induce cervical carcinogenesis.

The apparent role of folate in carcinogenesis in cervical tissue has stimulated investigations of polymorphisms in the folate metabolizing enzymes. As we know, Methylenetetrahydrofolate reductase (MTHFR) is a crucial enzyme that can regulate the metabolism of folate and methionine, both of which are important in DNA methylation and synthesis [17]. This occurs through the conversion of 5, 10-methyltetrahydrofolate to 5-methyltetrahydrofolate (1-carbon metabolism), which is a dominant circulating form of folate. The MTHFR gene is located on the short arm of chromosome 1 (1p36.3) and has several well-described single nucleotide polymorphisms (SNPs). Two common SNPs are known to affect enzyme function and have been shown to have clinical significance. The most common mutation is a C-to-T transition at nucleotide 677 (rs1801133, C677T) in exon 4, resulting in a substitution of alanine with valine that affects the catalytic domain of the enzyme, leading to the enzyme activity reduction [18]. Another common variant is an A-to-C transversion at position 1298 in exon 7 (rs1801131, A1298C), resulting in a substitution of glutamate with alanine at codon 429. This polymorphism also reduces the enzyme activity to a lesser extent [19].

Several studies had been designed to evaluate associations between MTHFR genotypes and cervical lesions, including cervical cancer, but the results were inconsistent because of different stages of cervical lesions and the combinatorial effects of other risk factors. Precancerous cervical lesions are classified according to the degree of cellular abnormality. The lowest grade of abnormality is CIN1, and CIN2 and CIN3 describe the progressive epithelial dysplasia leading to invasive cancer. Preinvasive lesions have also been classified in terms of squamous intraepithelial lesions (SILs) included low-grade squamous intraepithelial lesions (LSIL, including HPV infection and CIN1) and high-grade squamous intraepithelial lesions (HSIL, including CIN2 and CIN3). The majority of the case-control genetic studies revealed no association between cervical lesions and MTHFR SNPs [20]–[25]. But some evidences indicated that the MTHFR variants are positively associated with the cervical cancer risk [26]–[31]; some other evidence indicated that the MTHFR variants are inversely associated with the cervical cancer risk [32]–[35]. For example, one study reported that the MTHFR variant genotype may increase CIN and cervical cancer risk in women who had low-level folate status [26]. Another study suggested women with MTHFR polymorphism and low riboflavin status were significantly less likely to have HSIL than women without the polymorphism and high riboflavin status [33].

These inconclusive results may due to limited sample size, because any single study may be underpowered to detect the precise effects. In addition, there also may be the causes of different characteristics among studies, such as ethnicity, pathological history, sources of controls, and source of DNA for genotyping. Therefore, we have done a meta-analysis on association between MTHFR polymorphisms and cervical lesions using data obtained from the published case-control genetic studies. Our aim was to identify whether the MTHFR polymorphisms affect the susceptibility to SIL or cervical cancer by means of a large-scale meta-analysis. Furthermore, we wanted to summarize the effect size of the polymorphism associated with the susceptibility to the cervical lesions.

## Materials and Methods

### Search Strategy and Selection Criteria

The computer-based search strategy was comprehensively used to find eligible studies for this meta-analysis. Two investigators (Long, Yang) searched in the PubMed and Emase independently from inception to July 22, 2012, for the studies on the association between the MTHFR C677T polymorphism (rs1801133) and A1289C polymorphism (rs1801131) and the cervical lesions. Following Medical Subject Heading (MeSH) terms and/or text words were used in our search, such as for methylenetetrahydrofolate reductase (“MTHFR” or “methylenetetrahydrofolate reductase” or Methylenetetrahydrofolate Reductase AND (NADPH2)) with terms for genetic variations (“polymorphism” or “variation” or “mutation” or “Single Nucleotide Polymorphism” or Polymorphism, Single Nucleotide” or “SNPs” ) and terms for cervical lesions(“Uterine Cervical Cancer” or “Neoplasms, Cervix” or “Neoplasms, Cervical” or “Cervix Neoplasms” or “Cervix Cancer” or “Cervical Neoplasms” or “Cancer of the Uterine Cervix” or “Cancer of the Cervix” or “Cancer of Cervix” or “Uterine Cervical Neoplasms” or “Uterine Cervical Neoplasms” or “Uterine Cervical Dysplasia” or “Neoplasia, Cervical Intraepithelia” or “Intraepithelial Neoplasia, Cervical” or “Cervical Intraepithelial Neoplasms” or “Cervical Intraepithelial Neoplasia” or “cin” ). Meanwhile, China Biological Medicine Database (CBM) was also searched for the eligible studies. Full articles published in English or Chinese were considered to be eligible for our study. In addition, reference list of the original research articles and reviews were also manually searched.

The eligible studies must meet the following inclusion criteria: (1) Exploration of associations between the MTHFR genetic polymorphisms (including C677T or A1298C or both) and the susceptibility to cervical cancer or SIL; (2) A case-control study; (3) Provision of information on genotype frequencies of the MTHFR C677T and/or A1298C polymorphism(s) or sufficient data for the calculation. The exclusion criteria were as follows: (1) A review, case report, editorial, or comment; (2) A duplicated study; (3) Laboratory molecular or animal studies. If studies contained overlapping cases and/or controls, the largest study with extractable data was preferred.

Because the data included in this study was taken from literatures, written consent given by the patients and ethical approval acquired by certain committee were not needed in our meta-analysis.

### Data Extraction

According to the inclusion and exclusion criteria, extraction from each study was conducted independently by two authors (Long, Yang) and the consensus was achieved for all the data, which were as follows: the first author’s name, year of publication, source of controls, source of DNA for genotyping, country, ethnicity, goodness-in-fitness of Hardy-Weinberg Equilibrium (HWE) in the control group, histological stage of cervical lesions, numbers of cases/patients and controls, and distribution of genotypes in the case and control groups. The patients were recruited into the study regardless of whether they had a first-degree relative with cervical lesions. The controls were recruited regardless of whether they had other diseases, e.g., hysteromyoma. For studies with inadequate information, authors of those studies were contacted for further information by E-mail if possible.

### Statistical Analysis

Meta-analysis was performed and reported as described previously [36], [37]. Crude ORs with 95% CIs were computed to assess the strength of the correlation between the MTHFR C677T/A1298C polymorphisms and the susceptibility to cervical lesions. The pooled ORs were performed for the dominant model (aa+Aa *vs*. AA), recessive model (aa *vs*. Aa+AA) and additive model (A *vs*. a). Moreover, the pooled estimates were also calculated for the pair-wise comparisons (allele Aa *vs.* AA, and allele aa *vs.* AA). The above-mentioned A and a represented the major and the minor allele respectively. Taking consideration of possible between-study heterogeneity, a statistical test for heterogeneity was performed by the χ^2^ test or Fisher exact test when appropriate. *P*<0.10 or I^2^>50% indicated an obvious of the between-study heterogeneity, and OR (95% CI) was calculated by the random-effects model using the DerSimonian and Laird method; otherwise, the fixed-effects model was used by the Mantel-Haenszel method [38], [39]. Subgroup analyses were mainly conducted using the corresponding pathological history (ICC, SIL), geographic region (Asia, Europe, United States), ethnicity (Asian, Caucasian, mixed), source of controls (healthy persons, patients), and source of DNA for genotyping (blood, cervical cells or tissue sample), all of which were used to explore and explain the heterogeneity between the different studies.

The allele frequencies, at which the MTHFR C677T/A1298C polymorphisms occurred in each respective study, were determined by the allele-counting method. A chi-square test was used to determine whether the observed frequencies of the genotypes in the controls conformed to Hardy Weinberg-Equilibrium (HWE) expectations if genotype data were available. Sensitivity analyses were performed on stability of the results, namely, one case-control study omitted each time to reﬂect the inﬂuence of the individual data set on the pooled OR. Several methods were used to detect any probable publication bias. Asymmetry of the funnel plot indicated the possible publication bias. In addition, the Egger and Begg quantitative tests were also used, and *P*<0.05 was considered a statistical significance [40], [41].

All analyses were performed using the RevMan 5.0 program (Cochrane Library, UK) and the STATA package version 11.0 program (Stata Corporation, College Station, Texas, USA). All *P* values were two-sided. To ensure the reliability of data, two reviewers (Long, Yang) independently performed the data analysis using the statistics programs for the same results.

## Results

### Characteristics of Eligible Studies

**Figure 1:**
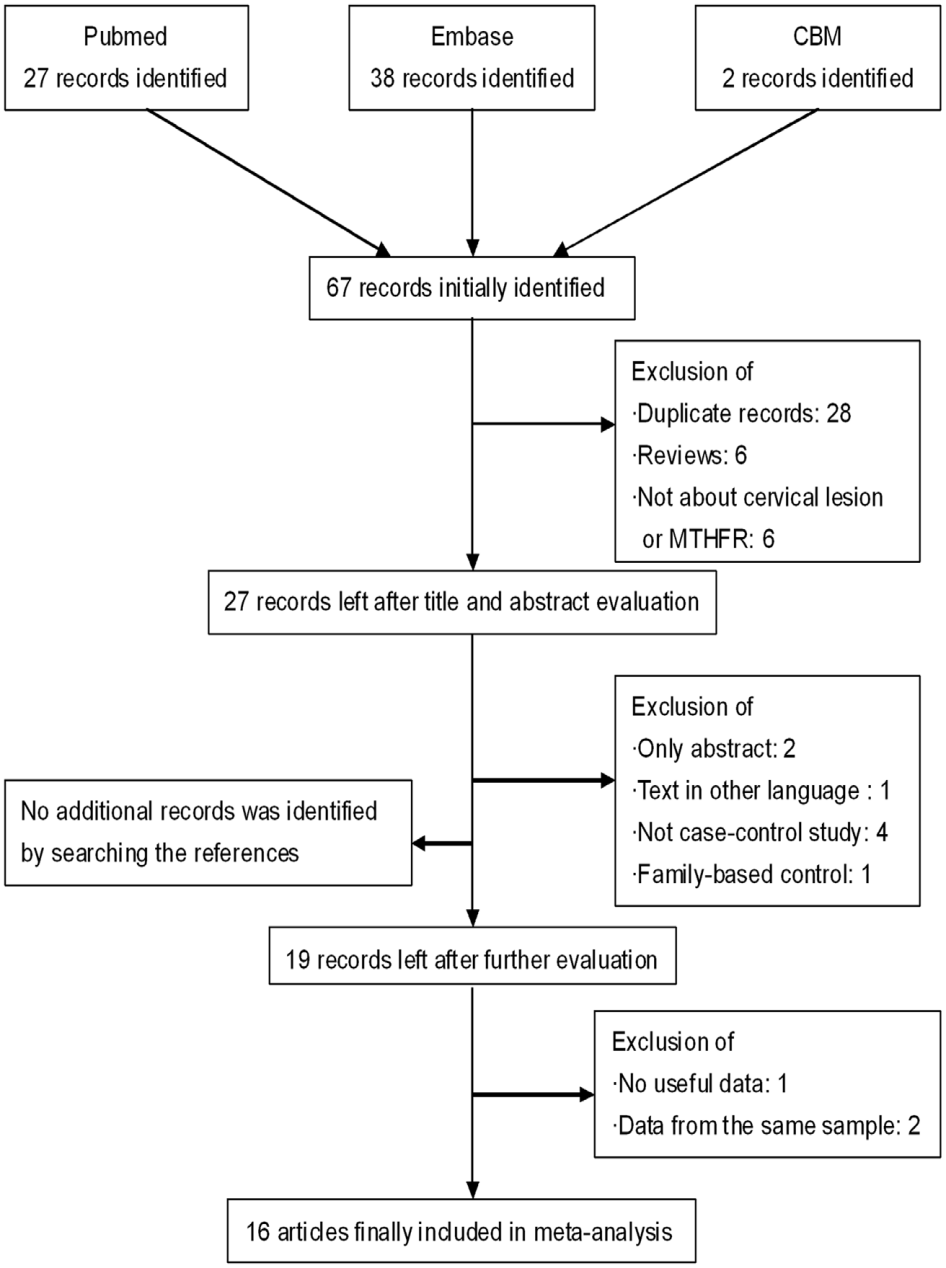
Flow diagram of the study selection process.

Detailed information for selecting eligible studies was showed in Figure 1. After comprehensively searching, 67 potentially-relevant publications were identified, and none of them were selected from the reference lists of the identified articles. After the careful selection, 16 eligible studies were finally included in our meta-analysis. Among them, 16 studies investigated the MTHFR C677T polymorphism with 3498 cases and 3594 controls and 5 studies investigated the MTHFR A1298C polymorphism with 1087 cases and 1202 controls. General characteristics of the included studies were evaluated for the association between variants and cervical lesions (Table 1, Table 2). For C677T, 11 studies recruited the controls from healthy persons; 1 study from hospital patients and 4 studies from both. 9 studies were performed in Asia; 4 studies performed in Europe; 3 studies performed in America. 5 studies talked about ICC; 3 studies talked about SIL and 8 studies talked about both. For A1298C, all 5 studies performed in Asian; 4 studies recruited controls from healthy persons and 1 study from both healthy persons and hospital patients. 1 study talked about ICC and 4 studies talked about both ICC and SIL. 14 of the studies presented NS (not significant) were conformed to Hardy Weinberg-Equilibrium (HWE) expectations (*P*>0.05). However, two of the studies [27], [35] presented NA (not available) were because we could not perform the HWE test for the subjects (either cases or controls) in those studies, for only the total number of the combined genotypes (CT/TT *vs.* CC or AC/CC *vs*. AA) were available. Therefore, this study was included in the analysis on the dominant model, not on other genetic models. Furthermore, the allele and genotype frequencies, at which the MTHFR C677T and the A1298C polymorphisms occurred in case and controls in each of the studies, were also summarized (Table 1, Table 2).

**Table 1:** Characteristics of the included case-control studies on the MTHFR C677T polymorphism in cervical lesions.

Firstauthor[reference]	Year	Sourceofcontrol	SourceofDNA	Country	Ethnicity	HWE	Histology	Samplesize		Case						Control					
								case	control	C	T	CC	CT	TT	CT+TT	C	T	CC	CT	TT	CT+TT
Prasad [20]	2011	Mixed	Blood	India	Asian	NS	ICC	62	125	119	5	57	5	0	5	240	10	116	8	1	9
Mostowska [21]	2011	Healthy persons	Blood	Poland	Caucasian	NS	ICC	124	168	194	77	56	59	9	68	219	117	69	81	18	99
Tong [26]	2011	Healthy persons	Blood	Korea	Asian	NS	LSIL	159	427	186	132	52	82	25	107	502	352	152	198	77	275
							HSIL	160	427	182	138	54	74	32	106	502	352	152	198	77	275
							ICC	146	427	171	121	53	65	28	93	502	352	152	198	77	275
Kohaar [22]	2010	Healthy persons	Tissue or cell	India	Asian	NS	HSIL	39	231	67	11	28	11	0	11	387	75	161	65	5	70
							ICC	164	231	273	55	113	47	4	51	387	75	161	65	5	70
Shekari [32]	2008	Healthy persons	Blood	India	Asian	NS	ICC	200	200	368	32	170	28	2	30	318	82	125	68	7	75
Nandan [27]	2008	Healthy persons	Blood	India	Asian	NA	SIL	80	77	NA	NA	34	NA	NA	46	NA	NA	53	NA	NA	24
							ICC	62	77	NA	NA	36	NA	NA	26	NA	NA	53	NA	NA	24
Piyathilake [33]	2007	Mixed	Blood	USA	Mixed	NS	HSIL	80	355	134	26	59	16	5	21	562	148	223	116	16	132
Zoodsma [34]	2005	Mixed	Blood	Netherlands	Caucasian	NS	HSIL	264	592	362	166	121	120	23	143	808	376	273	262	57	319
							ICC	636	592	944	328	357	230	49	279	808	376	273	262	57	319
Kang [23]	2005	Healthy persons	Blood	Korean	Asian	NS	ICC	79	74	86	72	27	32	20	52	92	56	30	32	12	44
Sull [28]	2004	Healthy persons	Blood	Korean	Asian	NS	LSIL	40	454	42	38	10	22	8	30	527	381	153	221	80	301
							HSIL	176	454	190	162	50	90	36	126	527	381	153	221	80	301
							ICC	246	454	261	231	73	115	58	173	527	381	153	221	80	301
Lambropoulos [24]	2003	Healthy persons	Tissue or cell	Greece	Caucasian	NS	LSIL	53	91	68	38	20	28	5	33	121	61	42	37	12	49
							HSIL	64	91	83	45	27	29	8	37	121	61	42	37	12	49
							ICC	21	91	30	12	11	8	2	10	121	61	42	37	12	49
Goodman [29]	2001	Hospital patients	Blood	USA	Mixed	NS	SIL	150	179	213	87	73	67	10	77	261	97	93	75	11	86
Piyathilake [30]	2000	Healthy persons	Tissue or cell	USA	Mixed	NS	LSIL	25	31	25	25	6	13	6	19	44	18	16	12	3	15
							HSIL	39	31	45	33	11	23	5	28	44	18	16	12	3	15
Agodi [35]	2010	Healthy persons	Cell	Italy	Caucasian	NA	SIL	123	66	NA	NA	118	NA	NA	5	NA	NA	55	NA	NA	11
Yang [25]	2011	Mixed	Blood	China	Asian	NS	SIL	38	382	60	16	23	14	1	15	530	234	182	166	34	200
							ICC	157	382	229	85	77	75	5	80	530	234	182	166	34	200
Ma [31]	2006	Hospital patients	Blood	China	Asian	NS	ICC	111	111	93	129	20	53	38	91	126	96	33	60	18	78

Abbreviations: HWE, Hardy-Weinberg Equilibrium; NA, not available; NS, not significant; LSIL, low-grade squamous intraepithelial lesion; HSIL, high-grade squamous intraepithelial lesion; ICC, invasive cervical cancer; SIL, squamous intra-epithelial lesion.

**Table 2:** Characteristics of the included case-control studies on the MTHFR A1298C polymorphism in cervical lesions.

First author[reference]	Year	Source of control	Source of DNA	Country	Ethnicity	HWE	Histology	Sample size		Case						Control					
								case	control	A	C	AA	AC	CC	AC+CC	A	C	AA	AC	CC	AC+CC
Tong [26]	2011	Healthy persons	Blood	Korea	Asian	NS	SIL	160	428	260	60	107	46	7	53	688	168	278	132	18	150
							HSIL	160	428	273	47	117	39	4	43	688	168	278	132	18	150
							ICC	148	428	235	61	89	57	2	59	688	168	278	132	18	150
Kohaar [22]	2010	Healthy persons	Tissue or cell	India	Asian	NS	HSIL	39	231	50	28	15	20	4	24	289	173	85	119	27	146
							ICC	164	231	199	129	58	83	23	106	289	173	85	119	27	146
Nandan [27]	2008	Healthy persons	Blood	India	Asian	NA	SIL	80	77	NA	NA	14	NA	NA	66	NA	NA	37	NA	NA	40
							ICC	62	77	NA	NA	20	NA	NA	42	NA	NA	37	NA	NA	40
Kang [23]	2005	Healthy persons	Blood	Korea	Asian	NS	ICC	79	84	132	26	55	22	2	24	141	27	58	25	1	26
Yang [25]	2011	Mixed	Blood	China	Asian	NS	SIL	38	382	62	14	24	14	0	14	606	158	237	132	13	145
							ICC	157	382	245	69	89	67	1	68	606	158	237	132	13	145

Abbreviations: HWE: Hardy-Weinberg Equilibrium; NA, not available; NS, not significant; LSIL, low-grade squamous intraepithelial lesion; HSIL, high-grade squamous intraepithelial lesion; ICC, invasive cervical cancer; SIL, squamous intra-epithelial lesion.

### Quantitative Synthesis

#### Association between the MTHFR C677T polymorphisms and cervical lesions

**Figure 2:**
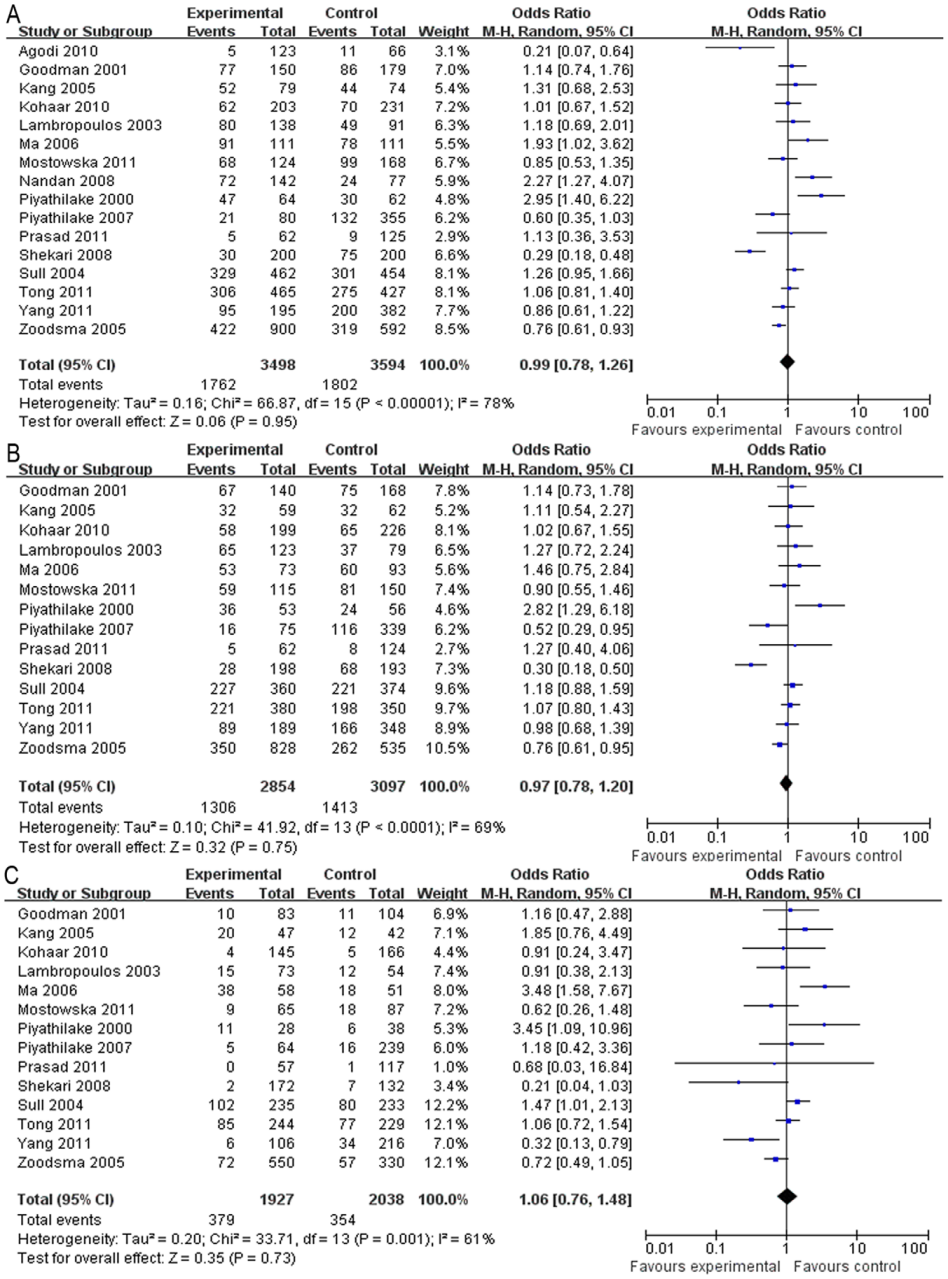
Forest plot describing the association between the C677T polymorphism and the risk of cervical lesions. (A) Meta-analysis in a random-effects model for CT+TT *vs.* CC (dominant model). (B) Meta-analysis in a random-effects model for CT *vs.* CC. (C) Meta-analysis in a random-effects model for TT *vs*. CC. Each study is shown by the point estimate of the OR (the size of the square is proportional to the weight of each study) and 95% CI for the OR (extending lines).

As for the C677T polymorphism, no association was found between the polymorphism and the susceptibility to cervical lesions in all the genetic models (Table 3, dominant model: OR = 0.99, 95% CI = 0.78–1.26, Figure 2A; recessive model: OR = 1.05, 95% CI = 0.80–1.38; additive model: OR = 0.97, 95% CI = 0.80–1.18,; CT *vs.* CC: OR = 0.97, 95% CI = 0.78–1.20, Figure 2B; TT *vs.* CC: OR = 1.06, 95% CI = 0.76–1.48, Figure 2C). The heterogeneity was significant in all the genetic models (*P*<0.05) and the random-effects model was used in the meta-analysis. The subgroup analysis of the C677T polymorphisms in the histological stages of the cervical lesions also revealed that the polymorphism was not associated with the risk of ICC or SIL in all the genetic models (Table 3). Although the subgroup analysis of C677T in the geographic regions revealed that no association was found between the C677T polymorphism and the cervical lesions in either the Asia or the America populations, the Europe population showed a significant inverse association in some genetic models (additive model: P = 0.006, OR = 0.83, 95% CI = 0.72–0.95; CT *vs*. CC: *P* = 0.05, OR = 0.83, 95% CI = 0.69–1.00; TT *vs*. CC: *P* = 0.05, OR = 0.73, 95% CI = 0.53–1.00). The heterogeneity was significantly reduced in the Europe populations in the recessive, additive, C/T *vs.* C/C, and T/T *vs*. C/C models.

**Table 3:** Pooled Analysis on Association between the MTHFR C677T polymorphism and the cervical lesion risk.

Genetic model	Number of study	Sample Size		Analysis	I^2^ (%)	P_h_	Test of Association		*P*(Publication bias test)	
		Case	Control	Model			P	OR(95%CI)	Begg’s test	Egger’s test
Total										
Dominant model	16	3498	3594	R	78	0.00	0.95	0.99 [0.78, 1.26]	0.558	0.626
Recessive model	14	3233	3451	R	51	0.01	0.75	1.05 [0.80, 1.38]	0.827	0.956
Additive model	14	6177	6902	R	79	0.00	0.79	0.97 [0.80, 1.18]	1.000	0.659
CT *vs.* CC	14	2854	3097	R	69	0.00	0.75	0.97 [0.78, 1.20]	0.443	0.490
TT *vs.* CC	14	1927	2038	R	61	0.00	0.73	1.06 [0.76, 1.48]	0.913	0.614
Pathological type										
ICC										
Dominant model	12	2008	2932	R	73	0.00	0.62	0.94 [0.72, 1.21]		
Dominant model*	11	1946	2855	R	73	0.00	0.44	0.90 [0.69, 1.18]		
Recessive model	11	1946	2855	R	59	0.00	0.96	1.01 [0.70, 1.45]		
Additive model	11	3915	5710	R	80	0.00	0.51	0.92 [0.73, 1.17]		
CT *vs.* CC	11	1731	2534	R	64	0.00	0.29	0.88 [0.69, 1.12]		
TT *vs.* CC	11	1229	1657	R	65	0.00	0.84	0.96 [0.62, 1.47]		
SIL										
Dominant model	11	1490	2916	R	71	0.00	0.54	1.09 [0.82, 1.45]		
Dominant modelˆ	9	1287	2773	R	52	0.04	0.51	1.08 [0.86, 1.35]		
Recessive model	9	1287	2773	F	0	0.79	0.80	1.03 [0.83, 1.27]		
Additive model	9	2574	5546	R	43	0.08	0.59	1.04 [0.90, 1.21]		
CT *vs*. CC	9	1123	2475	R	47	0.06	0.27	1.09 [0.94, 1.26]		
TT *vs.* CC	9	698	1609	F	0	0.45	0.36	1.11 [0.88, 1.40]		
Geographic area										
Asian										
Dominant model	9	1919	2081	R	80	0.00	0.71	1.07 [0.76, 1.49]		
Recessive model	8	1777	2004	R	65	0.00	0.74	1.08 [0.70, 1.66]		
Additive model	8	3242	4008	R	83	0.00	0.82	0.97 [0.71, 1.31]		
CT *vs.* CC	8	1520	1770	R	72	0.00	0.72	0.95 [0.70, 1.28]		
TT *vs*. CC	8	1064	1186	R	69	0.00	0.77	1.08 [0.65, 1.80]		
European										
Dominant model	4	1285	917	R	62	0.05	0.18	0.77 [0.52,1.13]		
Recessive model	3	1162	851	F	0	0.89	0.13	0.79 [0.58,1.07]		
Additive model	3	2347	1702	F	0	0.42	0.006	0.83 [0.72,0.95]		
CT *vs.* CC	3	1066	764	F	30	0.24	0.05	0.83 [0.69,1.00]		
TT *vs.* CC	3	688	471	F	0	0.82	0.05	0.73 [0.53,1.00]		
USA										
Dominant model	3	294	596	R	83	0.00	0.62	1.22 [0.56, 2.65]		
Recessive model	3	294	596	F	0	0.72	0.25	1.39 [0.79, 2.45]		
Additive model	3	588	1192	R	76	0.02	0.57	1.16 [0.70, 1.93]		
CT *vs.* CC	3	268	563	R	83	0.00	0.74	1.15 [0.50, 2.63]		
TT *vs.* CC	3	175	381	F	20	0.29	0.13	1.56 [0.88, 2.77]		

Dominant model: CT+TT *vs.* CC; Recessive model: TT *vs.* CC+CT; Additive model: T *vs.* C; R, Random-effects model; F, fixed-effects model; ICC: invasive cervical cancer; SIL, squamous intra-epithelial lesion; Dominant model*: one study [27] omitted; Dominant modelˆ: two studies [27], [35] omitted.

**Figure 3:**
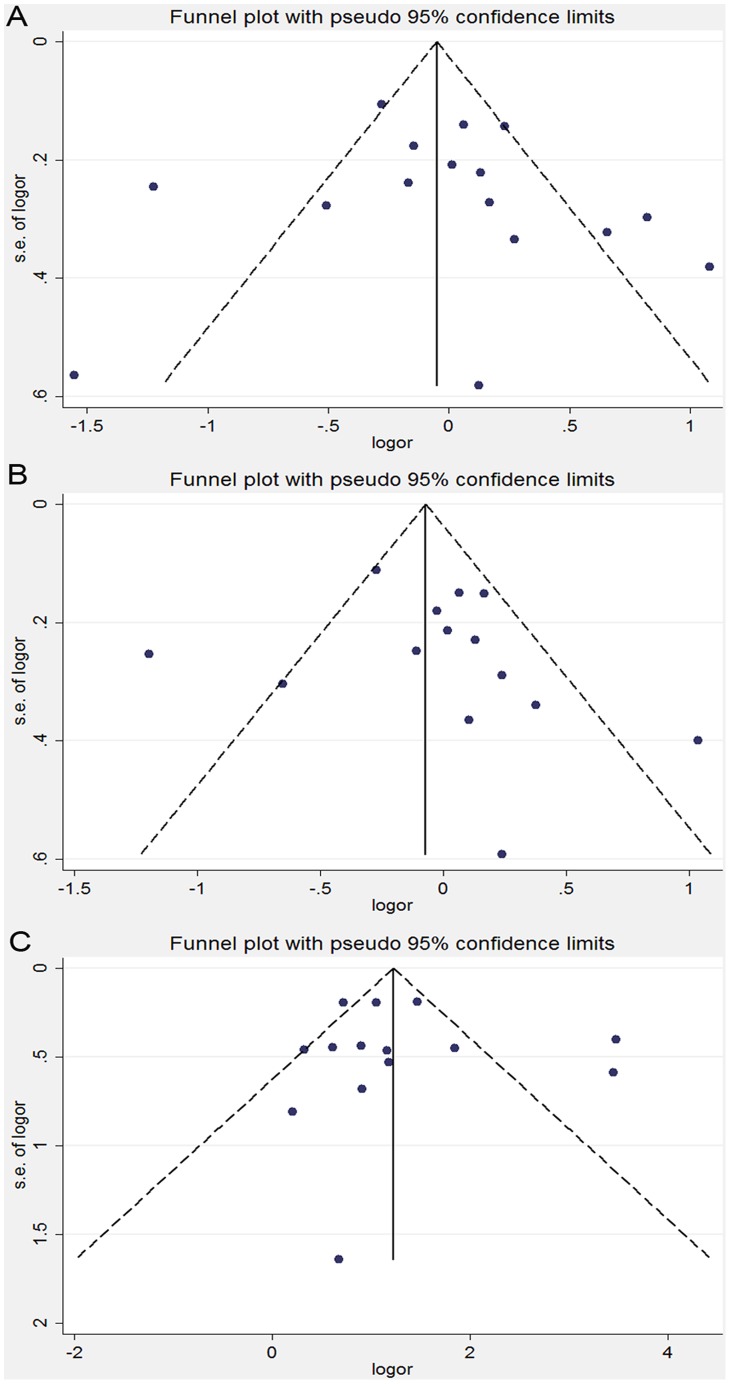
Funnel plot analysis on the detection of the publication bias for the C677T polymorphism. (A) Meta-analysis in a random-effects model for CT+TT *vs.* CC (dominant model). (B) Meta-analysis in a random-effects model for CT *vs.* CC. (C) Meta-analysis in a random-effects model for TT *vs*. CC. Each point represents an individual study for the indicated association. LogOR, natural logarithm of OR. Perpendicular line denotes the mean effect size.

In the sensitivity analysis, the overall association between the MTHFR C677T genotype and the cervical lesions was unchanged after an exclusion of the individual study, including two studies [27], [35], which lacked enough data to calculate if it conformed to HWE among the control group. Similar results were found in the sensitivity analyses on the association between the MTHFR C677T genotype and ICC or SIL, indicating that our results were statistically robust. No obvious publication bias was detected according to the shapes of the funnel plots for the C677T polymorphism in all the genetic models (Figure 3). Consistent results of the Egger’s and the Begg’s tests were also obtained in all the genetic models (Table 3). Moreover, neither the funnel plots nor the Begg’s or Egger’s test detected any obvious evidence for the publication bias in the subgroup analyses on all the genetic models (data not shown).

#### Association between the MTHFR A1298C polymorphisms and cervical lesions

**Figure 4:**
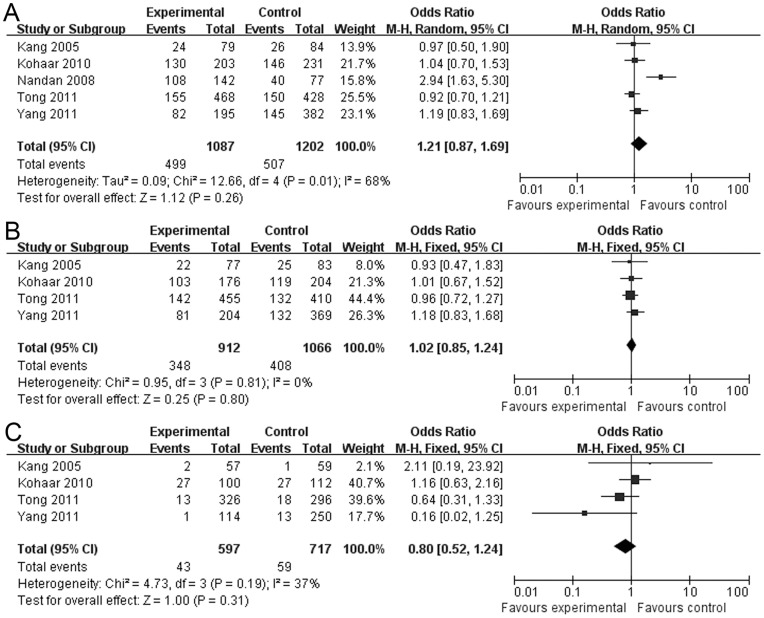
Forest plot describing the association between the A1298C polymorphism and the risk of cervical lesions. (A) Meta-analysis in a random-effects model for AC+CC *vs.* AA (dominant model). (B) Meta-analysis in a random-effects model for AC *vs.* AA. (C) Meta-analysis in a random-effects model for CC *vs.* AA. Each study is shown by the point estimate of the OR (the size of the square is proportional to the weight of each study) and 95% CI for the OR (extending lines).

As for the A1298C polymorphism, no association was found between the polymorphism and the cervical lesions in all the genetic models (Table 4, dominant model: OR = 1.21, 95% CI = 0.87–1.690, Figure 4A; recessive model: OR = 0.81, 95% CI = 0.54–1.23; additive model: OR = 0.98, 95% CI = 0.85–1.14; AC *vs.* AA: OR = 1.02, 95% CI = 0.85–1.24, Figure 4B; CC *vs.* AA: OR = 0.80, 95% CI = 0.52–1.24, Figure 4C). The heterogeneity was significant in the dominant model (I^2^ = 68%, *P* = 0.01) and the random-effects model was performed. However, there was no significant heterogeneity for the comparison of other genetic models (*P*>0.1) and the fixed-effects method was performed for our investigation. In the subgroup analysis, no association was found between the A1298C polymorphism and SIL. Interestingly, the investigation on the women with A1298C polymorphisms *vs.* no carriers showed a marginally increased susceptibility to ICC but no statistically significant difference in dominant model (*P* = 0.06, OR = 1.21, 95% CI = 0.99–1.49) and AC *vs.* AA (*P* = 0.09, OR = 1.21, 95% CI = 0.97–1.51).

**Table 4:** Pooled Analysis on Association between the MTHFR A1298C polymorphism and the cervical lesion risk.

Genetic model	Number of study	Sample Size		Analysis	I^2^ (%)	P_h_	Test of Association		*P*(Publication bias test)	
		Case	Control	Model			P	OR(95%CI)	Begg’s test	Egger’s test
Total										
Dominant model	5	1087	1202	R	68	0.01	0.26	1.21[0.87, 1.69]	0.462	0.290
Recessive model	4	945	1125	F	42	0.16	0.33	0.81[0.54, 1.23]	1.000	0.992
Additive model	4	1890	2250	F	0	0.81	0.82	0.98[0.85, 1.14]	1.000	0.587
AC *vs.* AA	4	912	1066	F	0	0.81	0.80	1.02[0.85, 1.24]	1.000	0.930
CC *vs.* AA	4	597	717	F	37	0.19	0.31	0.80[0.52, 1.24]	1.000	0.971
Pathological type										
ICC										
Dominant model	5	610	1202	F	0	0.63	0.06	1.21[0.99, 1.49]		
Recessive model	4	548	1125	R	51	0.10	0.46	0.67[0.24, 1.93]		
Additive model	4	1096	2250	F	0	1.00	0.43	1.07[0.90, 1.27]		
AC *vs.* AA	4	520	1066	F	0	0.62	0.09	1.21[0.97, 1.51]		
CC *vs.* AA	4	319	717	F	43	0.15	0.46	0.82[0.49, 1.38]		
SIL										
Dominant model	4	477	1118	R	83	0.00	0.49	1.28[0.63, 2.60]		
Recessive model	3	397	1041	F	0	0.85	0.43	0.78[0.42, 1.44]		
Additive model	3	794	2082	F	0	0.90	0.14	0.85[0.68, 1.06]		
AC *vs.* AA	3	382	983	F	0	0.75	0.25	0.85[0.65, 1.12]		
CC *vs.* AA	3	278	658	F	0	0.86	0.34	0.74[0.40, 1.38]		

Dominant model: CC+AC *vs.* AA; Recessive model: CC *vs.* AC+AA; Additive model: C *vs.* A; R, Random-effects model; F, fixed-effects model; ICC, invasive cervical cancer; ICC: invasive cervical cancer; SIL, squamous intra-epithelial lesion.

**Figure 5:**
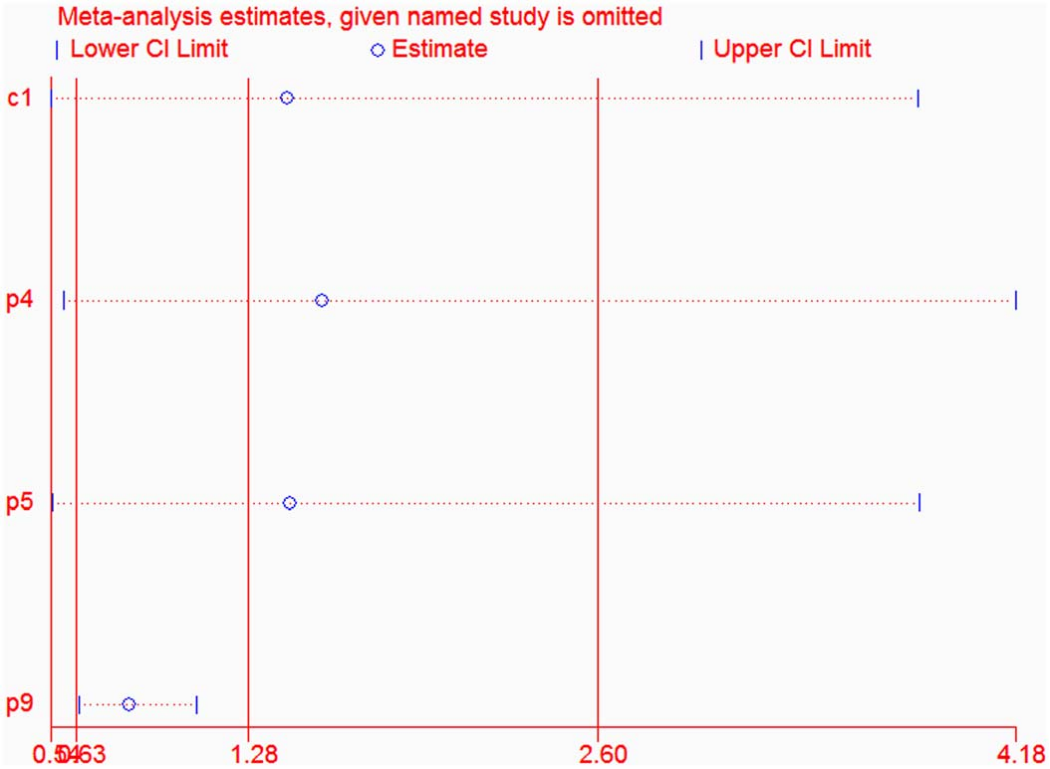
Influence analysis of the summary odds ratio coefficients on the association between the A1298C polymorphism and cervical cancer in dominant model. The results were computed by omitting each study (left column) in turn. Bars, 95% CIs.

**Figure 6:**
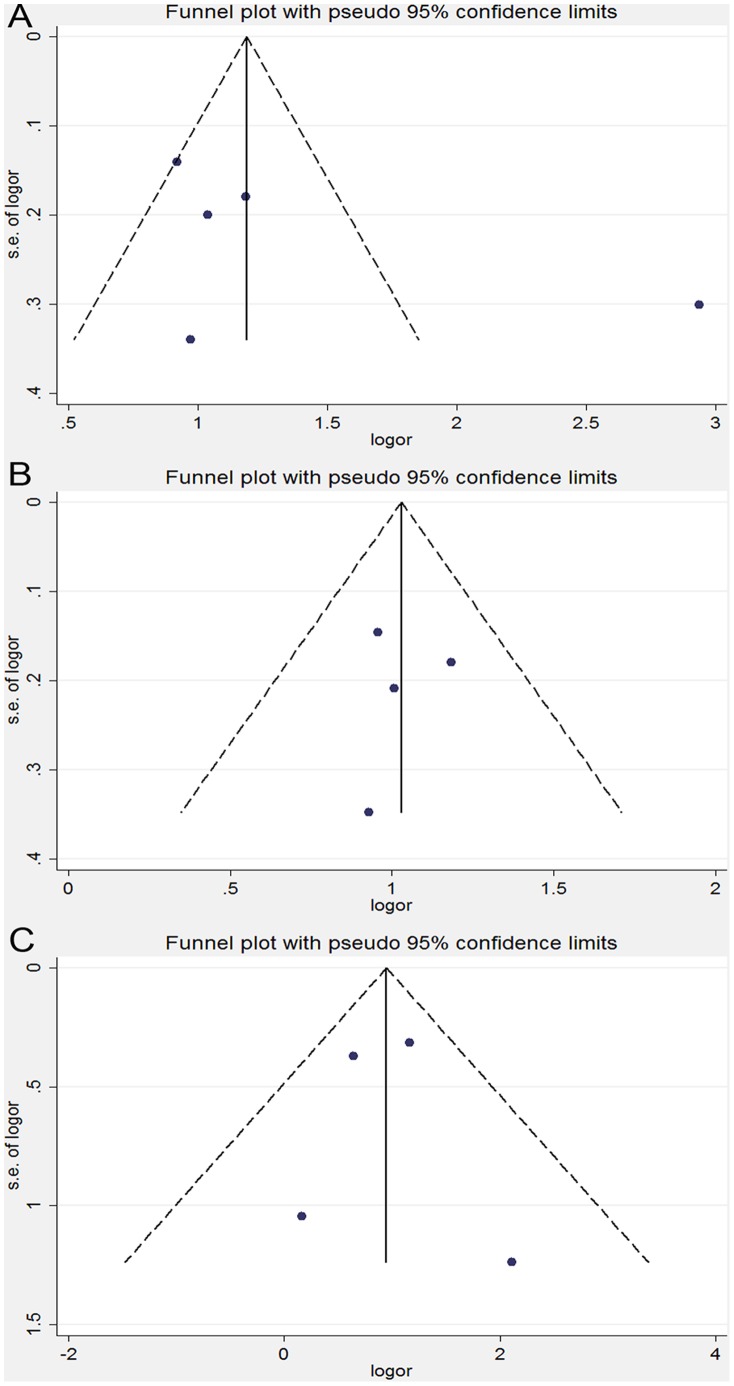
Funnel plot analysis on the detection of the publication bias for the A1298C polymorphism. (A) Meta-analysis in a random-effects model for AC+CC *vs*. AA (dominant model). (B) Meta-analysis in a random-effects model for AC *vs.* AA. (C) Meta-analysis in a random-effects model for CC vs. AA. Each point represents an individual study for the indicated association. LogOR, natural logarithm of OR. Perpendicular line denotes the mean effect size.

In the sensitivity analyses, the overall association between the MTHFR A1298C genotype and the cervical lesions was changed after an exclusion of one study [27] which lacked enough data to calculate if it conformed to HWE among the control group. However, the results of the sensitivity analysis on the cervical lesions were virtually unchanged after an exclusion of any other individual study (Figure 5). The shape of the funnel plots was symmetrical, which showed that no evidence was found for the publication bias among the studies (Figure 6). No publication bias was also detected according to the results of Egger’s and Begg’s tests (Table 4). Furthermore, neither the funnel plots nor the Begg’s and Egger’s tests found any obvious evidence for the publication bias in the subgroup analysis on all genetic models (data not shown).

## Discussion

As we know, HPV infection may be necessary but is not sufficient to cause cervical cancer. Other factors may play some important roles in this cancer development. For example, the nutritional factors may affect the persistence of HPV infection and thereby inﬂuence progression of early precancerous lesions to invasive cancer. Specifically, folate plays a key role in DNA synthesis, repair, and methylation, and this forms the basis of mechanistic explanations for a putative role for folate in cancer prevention. However, the effect of folate in these processes may be modulated by the genotype for the common C677T or A1298C variants of MTHFR, the homozygosity of which is associated with a lower level of the enzyme activity, lower plasma and red blood cell folate, and elevated plasma homocysteine [42], [43]. Several studies investigated the association between the MTHFR polymorphisms and the preinvasive cervical lesions or cervical cancer, but the results were not consistent. Thus, our meta-analysis could better evaluate association between the MTHFR C677T/A1298C polymorphisms and the susceptibility to cervical lesions. Our findings demonstrate that there was no association between them. To our knowledge, this is the first meta-analysis on association between MTHFR C677T/A1298C polymorphisms and susceptibility to cervical lesions, and the largest-scale meta-analysis examining the risk of cervical cancer.

As for the MTHFR C677T, most evidence points to decrease in the susceptibility to colorectal cancer and an increase in the susceptibility to esophagus and gastric cancer [44]–[48], but the effect on the cervical cancer susceptibility was not consistent. In our meta-analysis, no statistically significant difference was found in the frequency of the MTHFR C677T polymorphism in the patients with cervical lesions when compared with the controls. This finding was consistent with that of one previous meta-analysis [49]. However, 9 new studies [20]–[22], [25]–[27], [32], [33], [35] have been published since 2006 and all recruited in our study dramatically increased the case number of cervical lesion and controls with genetic information, which indicated that our results could be more reliable. In addition, multiple subgroup analyses made our meta-analysis more convincing too. We meta-analyzed the eligible case-control studies for C677T by geographic regions. No association was found between the C677T polymorphism and the cervical lesions in either in the Asian or in the American populations. However, a significant inverse association was found in the European population. Different genetic backgrounds or environmental conditions could explain the discrepancy. The meta-analysis also stratified by histological stages of cervical lesions showed that there was no association between the MTHFR C677T variants and cervical lesion development. To assess the effect of individual study on the overall meta-analysis estimate, we excluded one study at a time, and the exclusion of any single report did not change the significance of the final conclusion, which indicated that the outcomes were robust. Taken together, we could make a conclusion that cervical lesion were not primarily caused by genetically-determined enzymatic defects in the folate metabolic pathway, which might be different from the pathways supposed for colorectal or gastric carcinogenesis. The effect of those polymorphisms on the cervical cancer susceptibility seems to be further modulated by other cofactors such as infection with the HPV and smoking.

As for MTHFR A1298C, some studies reported a positive association with cervical lesions, which had only borderline significance [25]. More recent studies have revealed no association between the MTHFR A1298C and the cervical lesions [22], [23], [26], [27]. Our meta-analysis confirmed that there is no association between the A1298C polymorphism and cervical lesions, similar to that found by the subgroup analysis on the ethnic groups and the histological stages of cervical lesions. No association was found between the A1298C polymorphism and SIL, but the ICC showed a marginally positive association though with no statistically significant difference. This result suggested that a probably higher risk for cervical cancer was linked to the A1298C variants, implying their important role in later stages of cervical carcinogenesis but not in SILs. Sensitivity analyses revealed that the overall association between the MTHFR A1298C genotype and cervical lesions could be changed after excluding one study [27] which lacked sufficient data to calculate whether it conformed to HWE among or not in the control group. In contrast, the results were virtually unchanged after the exclusion of any other individual study. To sum up, it is possibly indicated that the study by Nandan et al. could be the main source of the observed heterogeneity across the studies in this meta-analysis. Alternatively, the study may had limitations or because of other unknown factors.

To some extent, several limitations of this meta-analysis should be addressed. One limitation of the present study was that the sample size of A1298C mutation involved is not big enough. We neen more original researches to make our conclusions more reliable and accurate. The studies on the A1298C variant had reported only 5 articles, and their participants were entirely Asians with no population variation in minor allele frequency. So, the subgroup meta-analysis on this gene polymorphism was not possible by race. Another limitation was that significant heterogeneity in the studies was mainly present in overall analyses and subgroup analyses. Though several possible sources of the between-study heterogeneity were investigated, including pathological history, geographic region, ethnicity, source of controls, and source of DNA for genotyping ethnicity (data not shown), none of them could sufficiently explain the heterogeneity. The effect estimates might depend on some unidentified sources of heterogeneity. Besides, part of the exposure information was still lacking in the available studies, E.g., HPV infection status, smoking status or nutritional status (particularly folate intake or level). Therefore, effects of environment exposure or lifestyle on association between MTHFR variants and cervical lesions could not be determined by this meta-analysis.

In summary, despite the above-mentioned limitations, the present study provides evidence that the MTHFR C677T and A1298C polymorphisms may not increase the susceptibility to cervical cancer development. However, our meta-analysis reveals a negative association between the MTHFR C677T mutations and cervical lesions, especially in the European populations. The marginal association between the MTHFR A1298C polymorphisms and the susceptibility for cervical cancer need to be further studied.

## Supporting Information

Table S1 **PRISMA checklist.**
(DOC)
